# A 7-gene signature predicts the prognosis of patients with bladder cancer

**DOI:** 10.1186/s12894-022-00955-3

**Published:** 2022-01-28

**Authors:** Fucai Tang, Zhibiao Li, Yongchang Lai, Zechao Lu, Hanqi Lei, Chengwu He, Zhaohui He

**Affiliations:** 1 Department of Urology, The Eighth Affiliated Hospital, Sun Yat-Sen University, Shennan Zhong Road #3025, Futian District, Shenzhen, 518033 Guangdong China; 2 The Third Clinical College of Guangzhou Medical University, Guangzhou, 511436 Guangdong China; 3 Department of Urology, The Seventh Affiliated Hospital of Sun Yat-Sen University, Shenzhen, Guangdong China; 4 Department of Urology, Sun Yat-Sen Memorial Hospital of Sun Yat-Sen University, Guangzhou, Guangdong China

**Keywords:** Bladder cancer, TCGA, GEO, Prognosis, Nomograms

## Abstract

The biomarkers have an important guiding role in prognosis and treatment of patients with bladder cancer (BC). The aim of the present study was to identify and evaluate a prognostic gene signature in BC patients. The gene expression profiles of BC samples and the corresponding clinicopathological data were downloaded from GEO and TCGA. The differentially expressed genes (DEGs) were identified by R software. Univariate Cox regression and the least absolute shrinkage and selection operator (LASSO) Cox regression were applied to construct the prognostic score model. A nomogram was established with the identified prognostic factors to predict the overall survival rates of BC patients. The discriminatory and predictive capacity of the nomogram was evaluated based on the concordance index (C‐index), calibration curves and decision curve analysis (DCA). A 7-gene signature (KLRB1, PLAC9, SETBP1, NR2F1, GRHL2, ANXA1 and APOL1) was identified from 285 DEGs by univariate and LASSO Cox regression analyses. Univariate and multivariate Cox regression analyses showed that age, lymphovascular invasion, lymphatic metastasis, metastasis and the 7-gene signature risk score was an independent predictor of BC patient prognosis. A nomogram that integrated these independent prognostic factors was constructed. The C-index (0.73, CI 95%, 0.693–0.767) and calibration curve demonstrated the good performance of the nomogram. DCA of the nomogram further showed that this model exhibited good net benefit. The combined 7-gene signature could serve as a biomarker for predicting BC prognosis. The nomogram built by risk score and other clinical factors could be an effective tool for predicting the prognosis of patients with BC.

## Introduction

Bladder cancer (BC) is a common tumour of the urinary system. In recent years, its incidence has shown an increasing trend, and BC patients have high recurrence and morbidity rates [1, 2]. It is estimated that there were more than 500,000 new cases of bladder cancer and approximately 200,000 BC-related deaths worldwide in 2018 [3]. According to the invasion of the muscle layer, BC can be divided into nonmuscle-invasive BC and muscle-invasive BC. Nonmuscle-invasive BC accounts for approximately 75% of bladder cancers. The 5-year survival rate of nonmuscle-invasive BC patients is high, but these patients are prone to relapse [4, 5]. Muscle-invasive BC accounts for approximately 25% of BCs. Among these muscle-invasive BC patients, the degree of progression is higher and the rate of tumour metastasis is greater than that for nonmuscle-invasive BC; in addition, the 5-year survival rate is only 50%, and a more thorough treatment strategy is needed [6, 7]. Therefore, it is of great clinical significance to investigate factors involved in the development and prognosis of BC. At present, the TNM staging system can predict the prognosis of patients and suggest treatment strategies. However, TNM staging is determined via clinical pathology, and it does not take into account the biological heterogeneity of tumours. Therefore, a more accurate and effective prediction model is needed to improve the risk prediction of patients, which could be beneficial for guiding individualized treatment.

In recent years, with the rapid development of tumour molecular biology, gene biomarkers have gradually gained attention. With the development of new technology and directions for screening and diagnosis of tumours, the search for tumour signatures with high sensitivity and specificity has been increasingly anticipated. However, the current gene biomarkers are mostly single gene biomarkers with limited accuracy, and the tumour prediction models developed from multiple signatures may provide more reliable and accurate evaluation than single clinical signatures [8]. In this study, the differentially expressed genes screened from Gene Expression Omnibus (GEO) and The Cancer Genome Atlas (TCGA) database were applied to establish a prognostic evaluation model for BC patients. This model integrates a gene signature and clinicopathological risk factors to provide an essential reference for prognostic evaluation and treatment in BC patients.

## Materials and methods

### Gene expression omnibus (GEO) and the cancer genome atlas (TCGA) databases

Gene expression profiles GSE7476, GSE37815 and GSE48075 were obtained from the GEO database (http://www.ncbi.nlm.nih.gov/geo/). GSE7476 contained 9 BC and 3 normal tissue samples. GSE37815 had 18 BC and 5 normal tissue samples. 72 BC samples were obtained from GSE48075, respectively. In addition, RNA-seq data and clinicopathologic characteristic data of BC patients and normal controls were obtained from the TCGA database (http://cancergenome.nih.gov/), including 414 BC and 19 normal tissue samples. The clinicopathologic characteristics and follow-up data included the patient's age, sex, diagnosis subtype, lymphovascular invasion, histologic grade, lymphatic metastasis, metastasis, and survival state and time. The exclusion criteria were as follows: (a) other cancer history; (b) lack of gene sequencing data or lack of clinical data;

### Data preprocessing and screening of differentially expressed genes

The Limma package in R software (version 3.5.3) was used to screen the differentially expressed genes (DEGs) in the gene expression profiles (GSE7476 and GSE37815). The edgeR package in R software was used to analyse the DEGs in the differential expression between BC and normal tissue from the TCGA and GEO. The threshold for DEGs was set to | log2 (fold change) |> 1, and an adjusted *P* value < 0.05 was considered statistically significant. These DEG results were combined and plotted by the Venn Diagram package in R software.

### Construction and assessment of the gene signature risk score model

The correlation between gene expression level and overall survival (OS) of BC patients was investigated by univariate Cox regression analysis using the survival package in R software, and *P* < 0.05 was considered statistically significant. Further, the above results were analysed by the least absolute shrinkage and selection operator (LASSO) and multivariate Cox methods using the glmmet package in R software. A gene signature risk score model for BC patients was constructed based on LASSO and multivariate Cox regression analysis results and was used to calculate the risk score for each BC patient. The risk score, which was calculated from the prognostic score model, divided all the patients into high- and low-risk groups by the median value. The Kaplan–Meier survival curve was performed for two groups by the survival package in R software. A time-dependent receiver operating characteristic (ROC) curve was performed for the risk score by the survivalROC package in R software, and the area under the curve (AUC) was calculated to judge the prediction results. The higher the AUC value is, the better the predictive performance of the model. Generally, AUC > 0.9 indicates a high accuracy, 0.7 < AUC ≤ 0.9 indicates a moderate accuracy, 0.5 < AUC ≤ 0.7 indicates a low accuracy, and AUC ≤ 0.5 indicates a lack of discriminating power.

### Construction and assessment of the predictive nomogram

Univariate Cox and multivariate Cox regression analyses were performed using the survival package in R software. A nomogram was established based on the above results to predict the 1-, 3-, 5-, and 8-year OS of BC patients by using the R software rms package. The calibration plots were used to evaluate the accuracy of the nomogram. The 45° line represented the best prediction value. The closer the curve was, the more ideal the result was. Decision curve analysis (DCA) was used to test the clinical value of this model [9].

## Results

### Screening of differentially expressed genes in bladder cancer

**Fig. 1:**
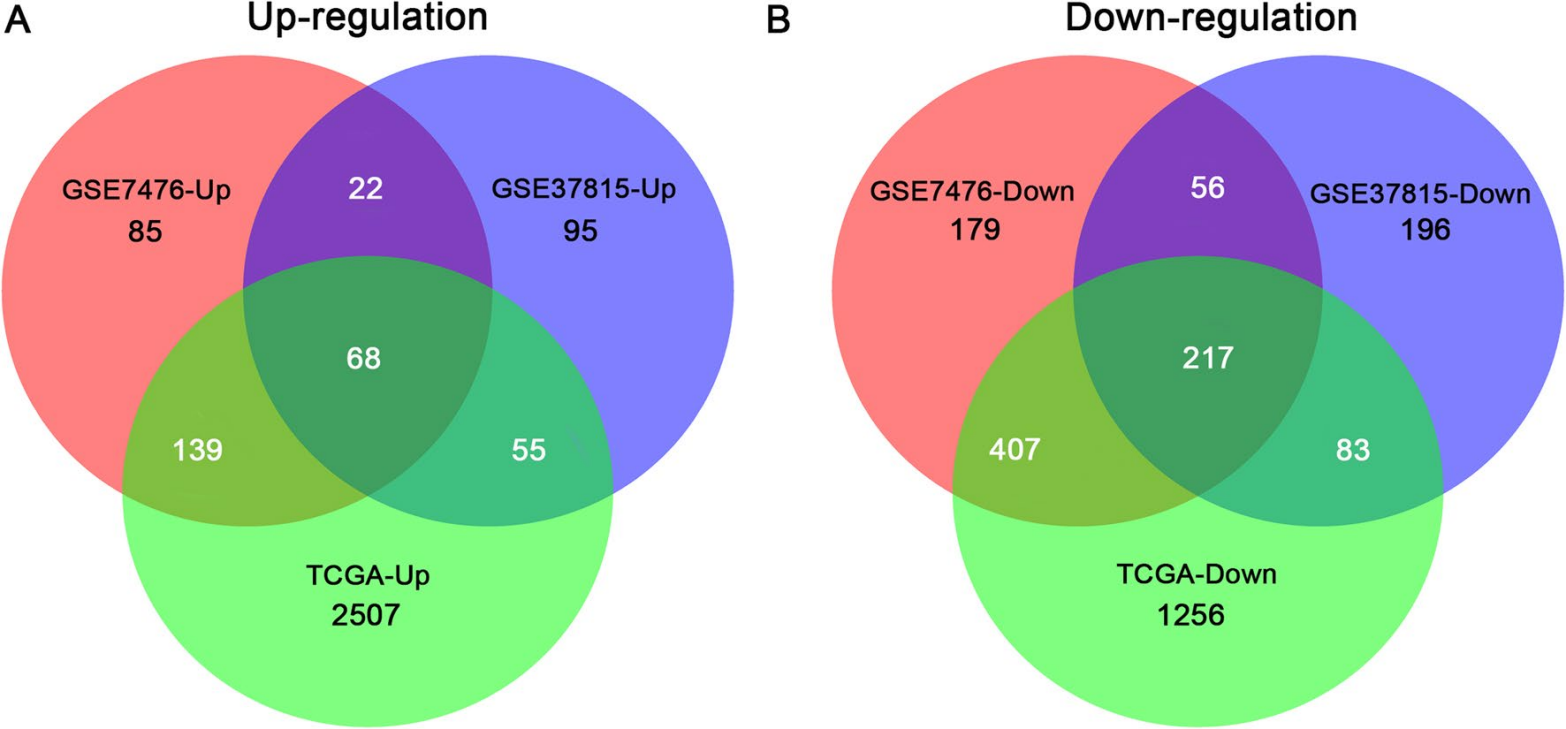
The Venn diagram of the common differentially expressed genes. **A** The Venn diagram indicates the number of upregulated DEGs in GSE7476, GSE37815 and TCGA. **B** The Venn diagram indicates the number of downregulated DEGs in GSE7476, GSE37815 and TCGA. The overlapping area indicates the cross-genes in GSE7476, GSE37815 and TCGA, respectively. TCGA, The Cancer Genome Atlas; DEGs, differentially expressed genes

The R difference analysis results showed that 314 genes were upregulated and 859 genes were downregulated in GSE7476, 240 genes were upregulated and 552 genes were downregulated in GSE37815, and 2769 genes were upregulated and 1963 genes were downregulated in the TCGA dataset. A total of 68 genes were upregulated and 217 genes were downregulated after merging these three results (Fig. 1).

### Identification of the 7-gene signature for survival prediction

**Fig. 2:**
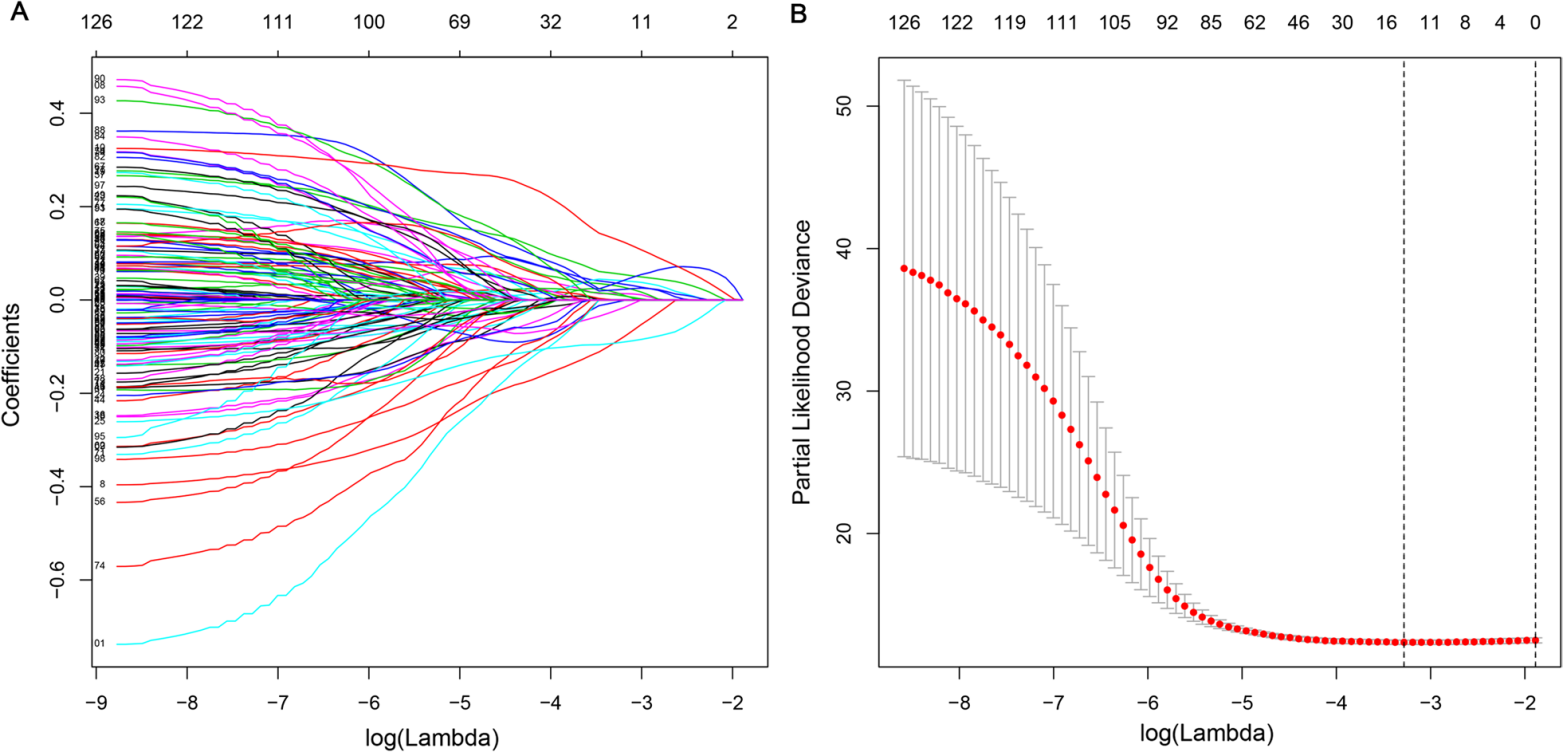
The LASSO analysis results: **A** The LASSO regression model using the ten-time cross-validation method to screen genes. **B** The 126 genes in the LASSO model regression coefficient profile. LASSO, least absolute shrinkage and selection operator

**Fig. 3:**
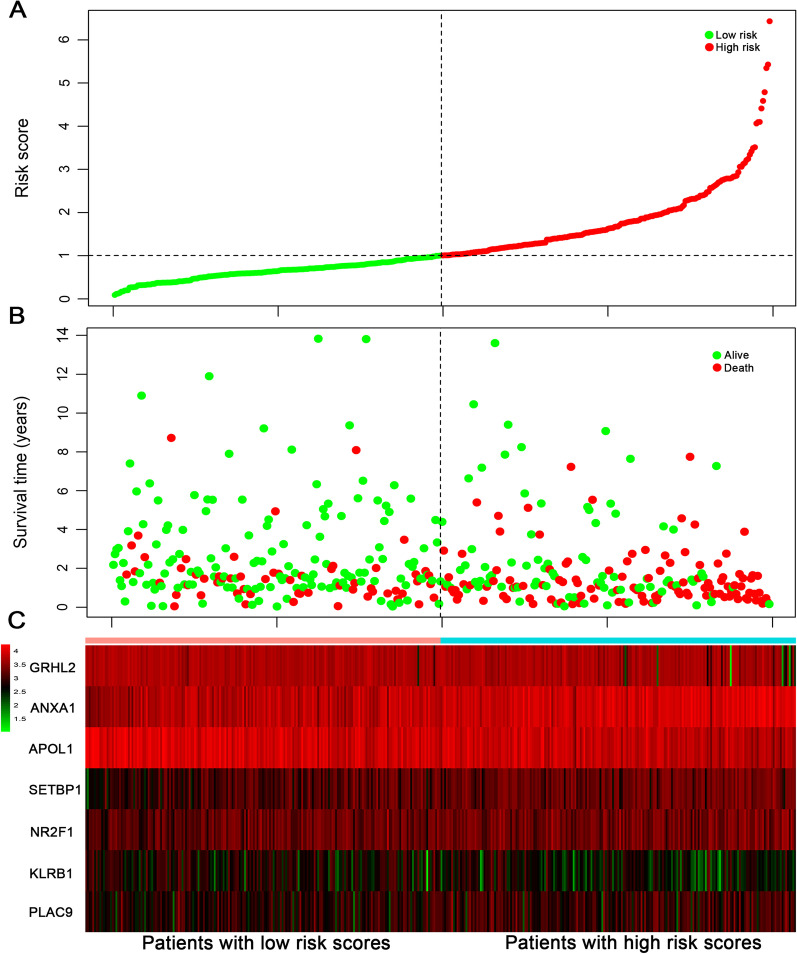
A prognostic risk model was constructed from the 7-gene signature, and the horizontal axis represents the high-risk and low-risk groups. **A** Distribution of the 7-gene signature risk score. The samples were grouped by the median risk score. The vertical line in the middle of the x-axis represents the high-risk and low-risk group boundaries: the left is the low-risk group, and the right is the high-risk group. Green represents the low-risk group, and red indicates the high-risk group. **B** The OS of BC patients in the high-risk and low-risk groups. Survival state and time corresponding to different risk scores in (**A**). Green indicates the patient was alive at follow-up, and red indicates already deceased. **C** Expression levels of 7 genes in the high-risk and low-risk groups. Green represents lower expression while red indicates higher expression. BC, bladder cancer; OS, overall survival

**Fig. 4:**
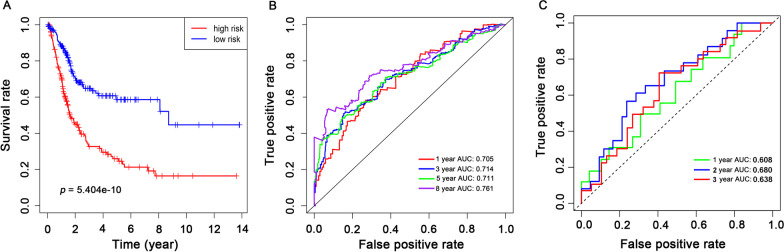
Kaplan–Meier curve and ROC curve analysis established according to the 7-gene signature prognostic model. **A** Kaplan–Meier curve OS comparison between the high-risk and low-risk groups. **B** ROC curve verification between the high-risk and low-risk groups of BC patients at 1, 3, 5 and 8 years. **C** ROC curve verification between the high-risk and low-risk groups of GSE48075 BC patients at 1, 2 and 3 years. ROC, receiver operating characteristic; OS, overall survival

A total of 126 prognostic-related genes were screened by univariate Cox regression analysis, and 7 key genes, including grainyhead-like 2 (GRHL2), annexin A1 (ANXA1), apolipoprotein L1 (APOL1), SET binding protein 1 (SETBP1), nuclear receptor subfamily 2 group F member 1 (NR2F1), killer cell lectin-like receptor subfamily B (KLRB1) and placenta-specific 9 (PLAC9), were identified using LASSO and multivariate Cox analysis (Fig. 2). The gene expression levels of PLAC9, SETBP1, NR2F1 and ANXA1 were positively correlated with the OS of BC patients, while KLRB1, GRHL2 and APOL1 were negatively correlated with the OS of BC patients. Based on the results of LASSO and multivariate Cox regression analysis, the risk score model was constructed. The risk score formula for this model was: (− 0.183 × expression value of KLRB1) + (0.106 × expression value of PLAC9) + (0.117 × expression value of SETBP1 + (0.115 × expression value of NR2F1) + (− 0.104 × expression value of GRHL2) + (0.220 × expression value of ANXA1) + (− 0.139 × expression value of APOL1). The 7-gene signature prognostic model was used to calculate the prognostic risk score of each patient. BC patients were divided into high-risk and low-risk groups according to the median score. The distribution of risk score, survival status and expression pattern in high-risk and low-risk groups are shown in Fig. 3. Mortality was significantly higher in the high-risk group than in the low-risk group. The predictive performance of the 7-gene signature prognostic model was assessed by using Kaplan–Meier curves and ROC curve AUC values. The Kaplan–Meier curve results showed that the prognosis of the two groups was significantly different (*p* < 0.001) and that the prognosis of the high-risk group was significantly lower than that of the low-risk group (Fig. 4A). The AUC values for this model (0.711, 0.714, 0.711, and 0.761 for the 1‐, 3‐, 5-, and 8‐year OS) demonstrated that the 7-gene signature prognostic model had good performance in predicting prognosis in patients with BC (Fig. 4B). GSE48075 was then used as the independent external test cohorts to validate the predictive prognostic power of the 7-gene signature. In the validation dataset, the 7-gene signature model showed good prediction efficiency with the area under the ROC curve (AUC) values at one year, two years, and three years equal to 0.608, 0.680 and 0.638, respectively (Fig. 4C).

### Establishment and evaluation of the nomogram

**Fig. 5:**
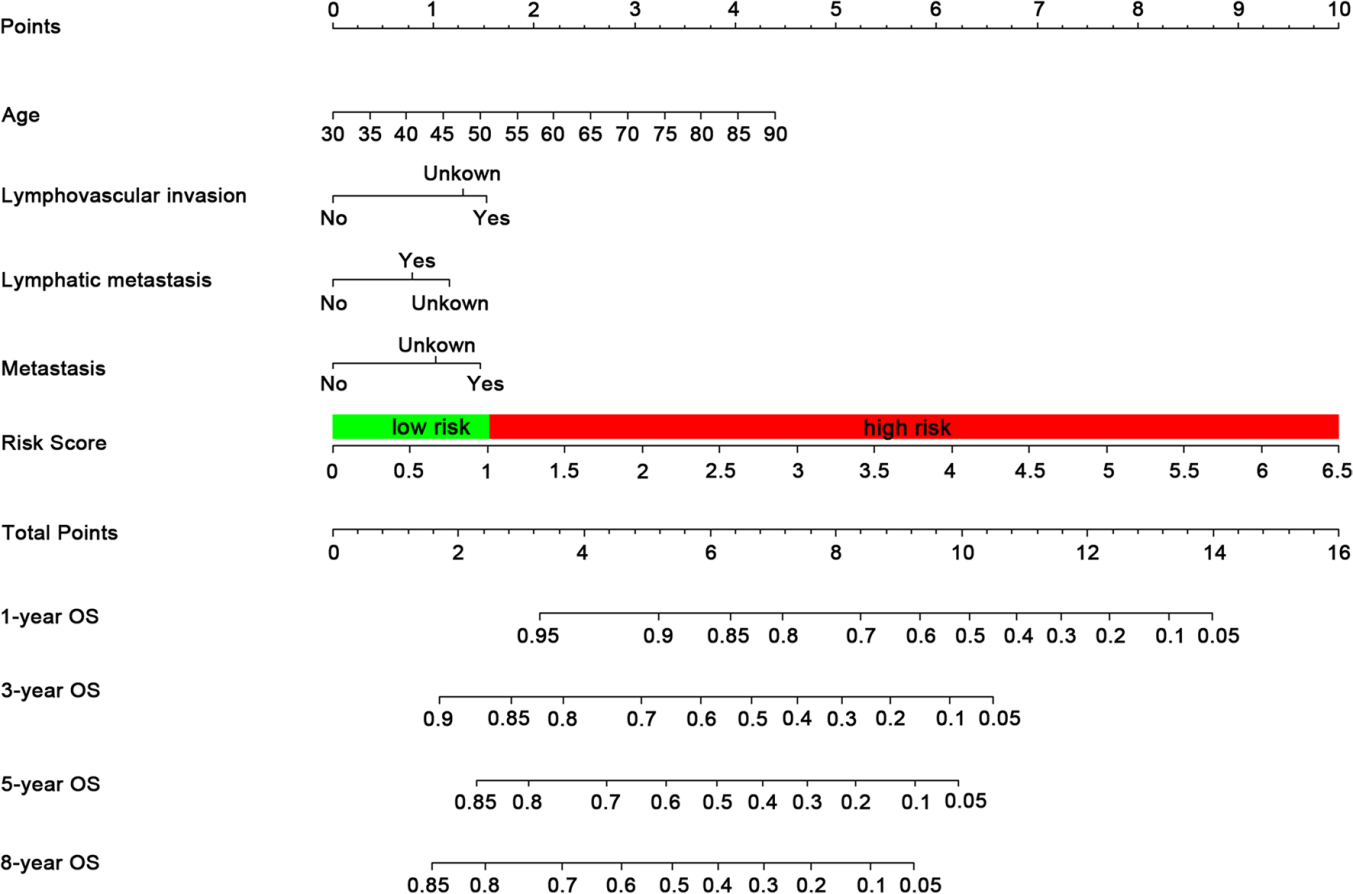
Nomogram for predicting probabilities of BC patients in 1-, 3-, 5- and 8-year OS. The nomogram is applied by adding up the points identified on the points scale for each variable. The total points projected on the bottom scales indicate the probability of 1‐, 3‐, 5‐ and 8-year OS. BC, bladder cancer; OS, overall survival

**Fig. 6:**
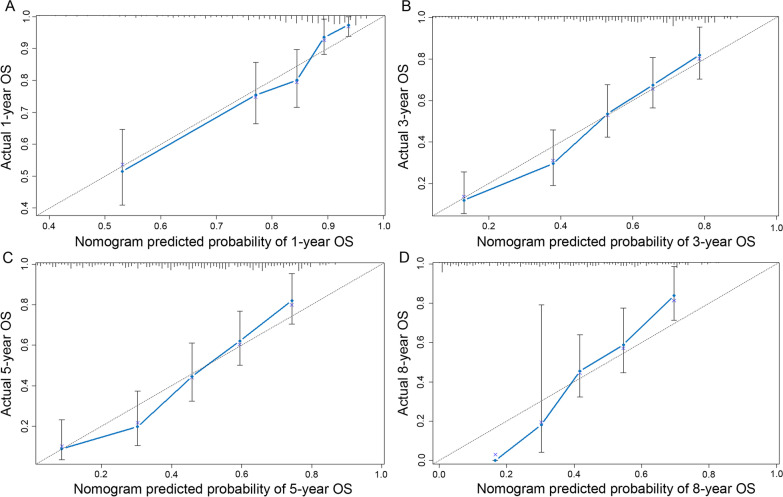
1-, 3-, 5- and 8-year OS calibration plots for BC patients based on the nomogram prognostic model. **A** The calibration plot for predicting BC patient 1‐year OS. **B** The calibration plot for predicting BC patient 3‐year OS. **C** The calibration plot for predicting BC patient 5‐year OS. **D** The calibration plot for predicting BC patient 8‐year OS. The Y‐axis indicates the actual survival of BC patients, and the X‐axis represents the nomogram‐predicted survival. The 45° line represents the best prediction value. The closer the curve is, the more ideal the result is. BC, bladder cancer; OS, overall survival

**Fig. 7:**
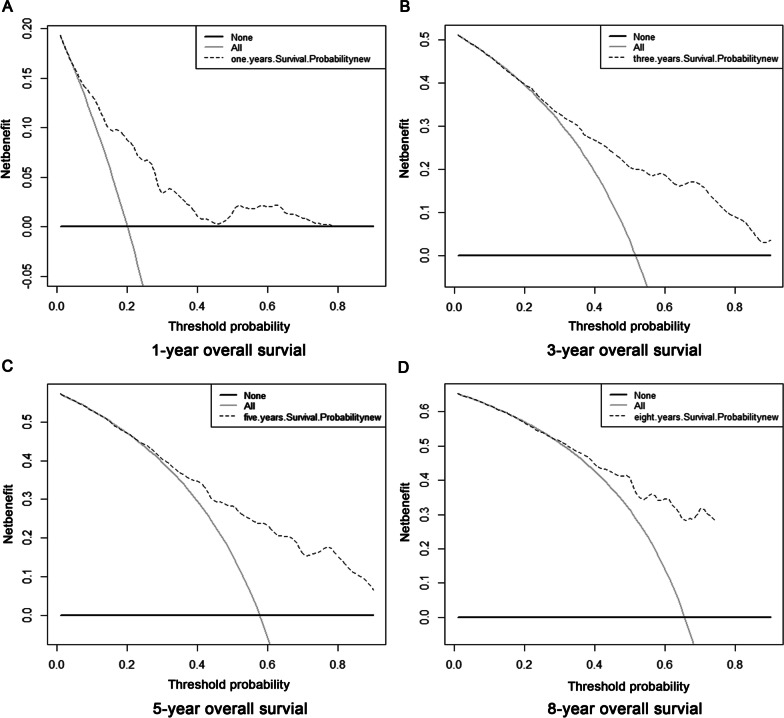
The 1-, 3-, 5- and 8-year OS from the DCA of BC patients based on the nomogram prediction model. **A** The DCA for evaluating BC patient 1-year OS; **B** The DCA for evaluating BC patient 3-year OS; **C** The DCA for evaluating BC patient 5-year OS; **D** The DCA for evaluating BC patient 8-year OS. The x-axis represents the threshold probability while the y-axis indicates the net benefit. The grey solid line represents the assumption that all patients reached the endpoint. And the black solid line represents the assumption that none of the patients reached the endpoint. The black dotted lines represent the nomogram. BC, bladder cancer; DCA, decision curve analysis; OS, overall survival

To assess whether the risk scoring model consisting of a 7-gene signature was independent of other clinical factors, univariate Cox and multivariate Cox regression analyses were performed according to clinicopathologic data and risk scores of BC patients. Univariate Cox and multivariate Cox results showed that age, diagnosis subtype, lymphovascular invasion, lymphatic metastasis, metastasis, and risk scores in BC patients were powerful and independent prognostic factors (Table 1). The nomogram was a statistical tool that could predict the overall probability of OS for BC patients with specific outcomes. Based on the results of multivariate Cox regression analysis, a nomogram was established to predict 1-, 3-, 5-, and 8-year OS (Fig. 5). The C-index of the nomogram for OS was 0.73 (CI 95%, 0.693–0.767). The calibration curve and DCA results showed that the nomogram predictions for 1-, 3-, 5-, and 8-year OS of BC patients had great accuracy and net benefit (Figs. 6, 7).

**Table 1:** Univariate and multivariate cox regression analysis of overall survival in the bladder cancer patients

Variables	N (%)	Univariate cox regression analysis		Multivariate cox regression analysis	
		HR (95%CI)	*P*	HR (95%CI)	*P*
*Sex*					
Female	106 (26.6%)	Reference		Reference	
Male	292 (73.4%)	0.853 (0.617–1.181)	0.340		
*Age*					
< 65	145 (36.43%)	Reference		Reference	
≥ 65	253 (63.57%)	2.064 (1.456–2.920)	< 0.001	2.002 (1.407–2.850)	< 0.001
*Diagnosis subtype*					
Non-papillary	270 (67.8%)	Reference		Reference	
Papillary	128 (32.2%)	0.682 (0.480–0.968)	0.032	0.885 (0.618–1.268)	0.507
*History of neoadjuvant treatment*					
N0	388 (97.5%)	Reference		Reference	
Yes	10 (2.5%)	1.140 (0.422–3.077)	0.796		
*Lymphovascular invasion*					
No	130 (32.7%)	Reference		Reference	
Yes	143 (35.9%)	2.195 (1.512–3.187)	< 0.001	1.671 (1.093–2.554)	0.018
Unkown	125 (31.4%)	1.629 (1.094–2.426)	0.016	1.585 (1.057–2.375)	0.026
*Histologic grade*					
High Grade	378 (95.0%)	Reference		Reference	
Low Grade	20 (5.0%)	2.890 (0.715–11.683)	0.137		
*Lymphatic metastasis*					
No	232 (58.29%)	Reference		Reference	
Yes	124 (31.16%)	2.293 (1.670–3.149)	< 0.001	1.645 (1.133–2.388)	0.009
Unkown	42 (10.55%)	1.692 (1.041–2.751)	0.034	1.926 (1.174–3.160)	0.009
*Metastasis*					
No	190 (47.7%)	Reference		Reference	
Yes	11 (2.8%)	3.299 (1.582–6.881)	0.001	1.632 (0.759–3.511)	0.210
Unkown	197 (49.5%)	1.551 (1.140–2.111)	0.005	1.437 (1.046–1.976)	0.025
*Risk*					
Low	199 (50.0%)	Reference		Reference	
High	199 (50.0%)	2.617 (1.909–3.588)	< 0.001	2.167 (1.556–3.018)	< 0.001

## Discussion

BC is one of the most common tumours in the urinary system. The global incidence rate of BC is increasing every year. It is one of the 10 most common cancers in men [10] and is a non-negligible hazard to human health. BC prognosis-related biomarkers have an important guiding role in diagnosis, prognosis and corresponding treatment methods. Therefore, it is one of the hot spots to find effective BC prognostic biomarkers. This study aimed to screen differentially expressed genes and construct a prognostic evaluation model of BC patients. The DEGs were obtained by R analysis of the GEO and TCGA databases in our study. Univariate Cox analysis was used for preliminary screening, and the results showed that 126 genes had significant differences that were related to the prognosis of BC. Seven genes (GRHL2, ANXA1, APOL1, SETBP1, NR2F1, KLRB1 and PLAC9) were identified to be independent prognostic factors for the prognosis of BC patients via LASSO and multivariate Cox regression analyses. These genes were used to establish a prognostic risk score model for BC patients to evaluate the OS of BC patients. The Kaplan–Meier curve and ROC curve demonstrated that the 7-gene signature prognostic model had a good prediction performance. The 7-gene signature risk score was confirmed to be independent of other clinicopathological features of BC patients by univariate Cox and multivariate Cox regression analyses. Further, the risk score and clinicopathological risk factors (age, lymphovascular invasion, lymphatic metastasis, metastasis) were used to establish a prognostic nomogram to predict the prognosis of patients at 1, 3, 5 and 8 years. The results of the calibration curve and DCA confirmed the reliability of the nomogram, which showed that the nomogram could help clinicians predict patient prognosis and provide guidance for patient rehabilitation assessment and treatment decision-making.

At present, although many genes have been identified as effective indicators for predicting the prognosis of BC patients [11–14], their predictive performance is relatively poor due to the limited number of screened genes as well as the lack of correlation with clinicopathological data. Compared with a single clinical gene biomarker, the integration of multiple related genes into a prognostic evaluation model can effectively improve the accuracy of prediction [15–17]. Similarly, the prognostic evaluation model of the 7-gene signature has displayed satisfactory predictive performance in this study. Among the 7 genes in the signature, PLAC9, SETBP1, ANXA1 and NR2F1 were negatively correlated with OS in BC patients. PLAC9 is a placental protein that is upregulated in embryonic expression (weeks 8 to 9) [18]. Ouyang C et al. reported that overexpression of PLAC9 induces G2 and M phase arrest in cell division and inhibits cell growth [19], but it has not been reported in bladder cancer. It might be necessary to further explore the specific role of PLAC9 in bladder cancer in the future. SETBP1 has been established as an important diagnostic marker for bone marrow malignancies, and its expression is closely related to the prognosis of patients. Studies have shown that it plays a key role in tumour invasion and rapid evolution [20]. Additionally, SETBP1 inhibits the expression of tumour protein phosphatase 2A (PP2A) [21] and regulates cell proliferation [22], suggesting that SETBP1 may also play a regulatory role in tumorigenesis and progression. ANXA1 functions to regulate a variety of cell biological behaviours, including membrane aggregation, phagocytosis, cell proliferation and tumorigenesis [23]. Reports have shown that ANXA1 is related to the recurrence and drug resistance of BC, and its expression level was positively correlated with T phase [24]. ANXA1 was shown to play an important role in the development of high-grade BC [25]. Our findings also demonstrated that high expression of ANXA1 is related to the occurrence and development of bladder cancer and affects the prognosis of BC patients. NR2F1 regulates the progression of cell differentiation, cancer progression, and central and peripheral neurogenesis [26]. Kikuchi-Koike R et al. indicated that NR2F1 is highly connected with the high-risk recurrence of breast cancer [27]. High NR2F1 expression has a strong effect on increasing lung metastatic potential. Additionally, the overexpression of NR2F1 was reported to enhance invasion and metastasis in salivary adenoid cystic carcinoma [28]. At present, the role of NR2F1 in BC has not been reported. In this study, we showed for the first time that NR2F1 is highly expressed in BC and is closely related to the prognosis of BC patients. However, the specific regulation of NR2F1 in BC still needs further exploration. The other three genes, GRHL2, APOL1 and KLRB1, were positively correlated with the OS of BC patients. GRHL2 enhances the effect of carcinogenesis and promotes tumour cell proliferation. It can also promote the growth of oral squamous cell carcinoma, colorectal cancer and hepatocellular carcinoma [29]. However, more reports have shown that GRHL2 can inhibit epithelial-mesenchymal transition to inhibit cell migration [30] and cancer metastasis [31]. Additionally, the overexpression of GRHL2 can effectively inhibit tumour invasion and migration in gastric and breast cancer [30, 32]. In this study, we confirmed that GRHL2 is significantly expressed at low levels in BC and has a positive regulatory effect on the prognosis of BC patients. GRHL2 may be an effective prognostic marker for BC. APOL1 is mainly active in the kidney [33], and its high expression can promote the occurrence of kidney disease [34]. In the study of Gutiérrez OM et al., the mortality rate of patients with a high-risk APOL1 genotype was significantly lower than that of patients with low-risk genotypes in the prognostic review cohort of chronic kidney disease [35]. However, there are few reports about the effect of APOL1 on BC, and further research is still needed. KLRB1 is involved in the coding of NKRP1A/LLT1, which triggers the activation of T cells and B cells and can be used in the treatment of cancer [36], and its interaction with LLT1 can inhibit natural killing (NK) cell cytotoxicity [37]. Many studies have shown that a high level of LLT1 expression can significantly restrain NK cells and enhance tumour immune escape, as knockdown of LLT1 enhances NK cell-mediated lysis of tumour tissues [38–40]. These results combined with our results demonstrate that KLRB1 is expressed at low levels in BC and is positively correlated with OS in BC patients. Because KLRB1 is capable of eliciting spontaneous antitumour immune responses, it may be a promising potential target for BC immunotherapy. Further experiments are needed to verify our hypothesis.

To better apply this model for predicting the OS of BC patients, the risk score was combined with other independent prognostic factors (age, lymphovascular invasion, lymphatic metastasis, metastasis) to establish a nomogram to predict the OS of BC patients at 1 year, 3 years, 5 years, and 8 years. The C-index, calibration curve and DCA results showed the good performance and clinically usefulness of the nomogram. These results showed that the nomogram established by combining these independent prognostic factors was an effective and accurate tool for predicting the OS of BC patients. Additionally, this nomogram played an important role in both short-term and long-term observation studies, so it might be helpful for patient consultations, clinical decision-making and determining follow-up arrangements for BC patients.

However, our research still has some limitations. First, most of the patients in the TCGA database were white or black. The racial diversity was limited, so the results may be biased towards these groups. Second, most of the selected genes were not previously reported in BC or lacked research in BC; therefore, further experimental validation was the direction of our research. Finally, the nomogram for predicting the OS of BC patients, which was based on the TCGA database, was only established and validated in a single data set, lacking further validation with external BC data. Therefore, corrections according to future verification studies in multiple different data sets are required.

## Conclusion

In summary, using univariate Cox, LASSO and multivariate Cox analyses, we identified seven key genes (GRHL2, ANXA1, APOL1, SETBP1, NR2F1, KLRB1, and PLAC9) and constructed a 7-gene signature prognostic model for BC patients. We also combined the 7-gene signature risk score with age, lymphovascular invasion, lymphatic metastasis and metastasis to construct a nomogram for prognostic evaluation. This nomogram could provide an important reference for prognostic evaluation and treatment decision-making for BC patients. However, the 7-gene signature in the BC patient prognosis assessment model still needs more clinical validation before it can truly be applied in clinical practice.

## Data Availability

The datasets of gene expression profiles for bladder cancer (GSE7476, GSE48075 and GSE37815) are available in the GEO on the NCBI website (http://www.ncbi.nlm.nih.gov/geo). The mRNA-Seq data and clinical follow-up data associated with the BC and para-bladder cancer tissues samples were downloaded from the TCGA (https://cancergenome.nih.gov/).

## Abbreviations

BC: Bladder cancer
GEO: Gene expression omnibus
TCGA: The cancer genome atlas
DEGs: Differentially expressed genes
LASSO: Least absolute shrinkage and selection operator
ROC: Receiver operating characteristic
C‐index: Concordance index
DCA: Decision curve analysis
OS: Overall survival
AUC: Area under the curve
NK cell: Natural killing cell
